# Carbon Quantum Dots Encapsulated Molecularly Imprinted Fluorescence Quenching Particles for Sensitive Detection of Zearalenone in Corn Sample

**DOI:** 10.3390/toxins10110438

**Published:** 2018-10-28

**Authors:** Manyu Shao, Ming Yao, Sarah De Saeger, Liping Yan, Suquan Song

**Affiliations:** 1 College of Veterinary Medicine, Nanjing Agricultural University, Nanjing 210095, China; shaomanyu@outlook.com (M.S.); 2017807151@njau.edu.cn (M.Y.); 2 Centre of Excellence in Mycotoxicology and Public Health, Ghent University, Ottergemsesteenweg 460, 9000 Ghent, Belgium; sarah.desaeger@ugent.be

**Keywords:** molecularly imprinted fluorescence quenching particles, carbon quantum dots, non-hydrolytic sol-gel, zearalenone, fluorescence quenching

## Abstract

An eco-friendly and efficient one-step approach for the synthesis of carbon quantum dots (CDs) that encapsulated molecularly imprinted fluorescence quenching particles (MIFQP) and their application for the determination of zearalenone (ZEA) in a cereal sample are described in this study. CDs with high luminescence were first synthesized, and then encapsulated in the silica-based matrix through a non-hydrolytic sol-gel process. The resulting ZEA-imprinted particles exhibited not only an excellent specific molecular recognition of ZEA, but also good photostability and obvious template binding-induced fluorescence quenching. Under the optimized conditions, the fluorescence intensity of MIFQP was inversely proportional to the concentration of ZEA. By validation, the detection range of these fluorescence quenching materials for ZEA was between 0.02 and 1.0 mg L^−1^, and the detection limit was 0.02 mg L^−1^ (S/N = 3). Finally, the MIFQP sensor was successfully applied for ZEA determination in corn with recoveries from 78% to 105% and the relative standard deviation (RSD %) was lower than 20%, which suggests its potential in actual applications.

## 1. Introduction

Zearalenone (ZEA) contaminates cereals and derived products across the world [1]. Toxic effects of ZEA include infertility, teratogenesis, neurotoxicity, carcinogenicity, and abortion [2,3]. Therefore, the International Agency for Research on Cancer (IARC) has listed ZEA as a group III carcinogen [4], and the increasing awareness of mycotoxins has increased the need for the accurate and reliable analysis of ZEA in cereal products.

Over the past few years, various methods, including thin-layer chromatography (TLC) [5], the enzyme-linked immunosorbent assay (ELISA) [6], and high-performance liquid chromatography (HPLC) based instrumental methods have been developed for ZEA detection [7,8,9]; however, these methods have some unavoidable limitations. For example, TLC lacks selectivity or sensitivity in complex matrices [10]; chromatographic-based methods need expensive instruments and professional operators [11]; and immunochemical techniques, such as ELISA, need highly specific antibodies, which are difficult to produce and conserve.

Molecularly imprinted polymers (MIPs) are synthetic highly cross-linked polymers, which form the specific cavities that are complementary to the size and shape of the template molecules during the copolymerization [12,13,14,15,16,17]. The resulting polymer can specifically rebind the template molecules in suitable solvents [18,19]. So far, there are two major preparation strategies. One is the silica-based system and the other is the organic-polymer-based system. The organic-polymer-based system has been widely used due to the availability of different monomers and its excellent stability in different pH environments. However, the polymers prepared using this method may shrink or swell in different solvents, which certainly affects their recognition of the template [20]. Silica-based monoliths may show good solvent resistance. However, these materials are often synthesized via hydrolytic sol-gel technology [21,22], which may crack or shrink during drying. Therefore, a feasible strategy to produce silica-based MIPs using the non-hydrolytic sol-gel (NHSG) technique has been developed, which can not only overcome the above mentioned shortcomings of the MIPs, but also decrease non-selective absorbance [23].

Recently, fluorescence-based sensing has received great attention for its application in biosensors owing to its low cost, excellent sensitivity, and fast response [24]. Among these sensory materials, quantum dots (QDs) have been widely used [25,26,27]. However, due to the usage of poisonous heavy metals in their synthesis, QDs can threaten human health and pollute the environment. Therefore, an alternative to QDs, new luminescent materials, such as graphene quantum dots (GQDs) and carbon quantum dots (CDs), have received more attention due to their high water solubility, low toxicity, easy functionalization, and excellent biocompatibility [28,29,30,31,32]. Because CDs can be prepared by the pyrolysis of organic molecules subjected to high temperatures, while the precursors of GQDs are mainly graphene-based materials, CDs is more popular in fluorescence-based sensing [33]. However, CDs are rarely applied in the food safety testing field [34,35]. The main obstacle lies in the immobilization of CDs in the polymers, which allows the permeation of analyte while avoiding the leaching of CDs. Therefore, silica-based MIPs may be a promising matrix that could efficiently protect the photoluminescent (PL) properties of CDs, without affecting the binding of the target molecules. However, in order to intensify the interaction between the silica matrix and CDs, the CDs should be previously pretreated with organosilane, which means that the preparation process of such polymers requires at least two steps.

**Figure 1:**
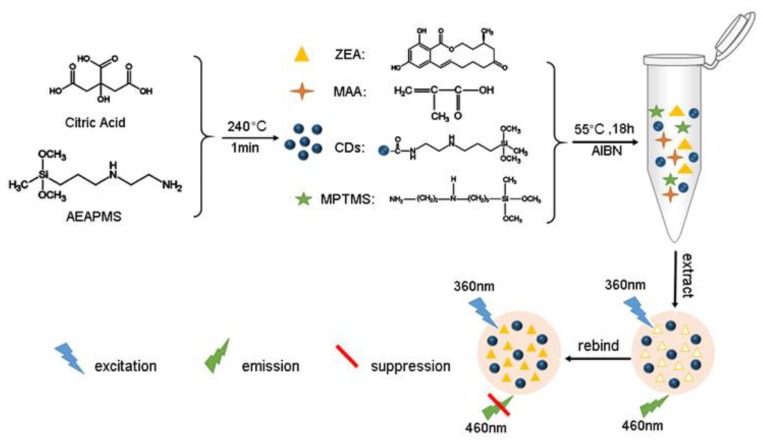
Preparation of MIFQP.

In this study, a novel eco-friendly fluorescence quenching material has been developed as a ZEA sensor through an efficient one-step NHSG molecular imprinting process. The synthesis of MIFQP is demonstrated in Figure 1. This polymer integrates the accurate selectivity of MIPs with the high fluorescence (FL) stability of CDs. In the sensor, CDs work as antennas for sample recognition and signal amplification, and polymers provide specific binding sites for ZEA. When ZEA is present in the sample, the FL of CDs is blocked. The degree of FL quenching is proportional to ZEA concentration. Importantly, with these materials, the simple, sensitive, and fast detection of ZEA in a corn sample can be realized.

## 2. Results and Discussion

### 2.1. Preparation and Characterization of CDs

**Figure 2:**
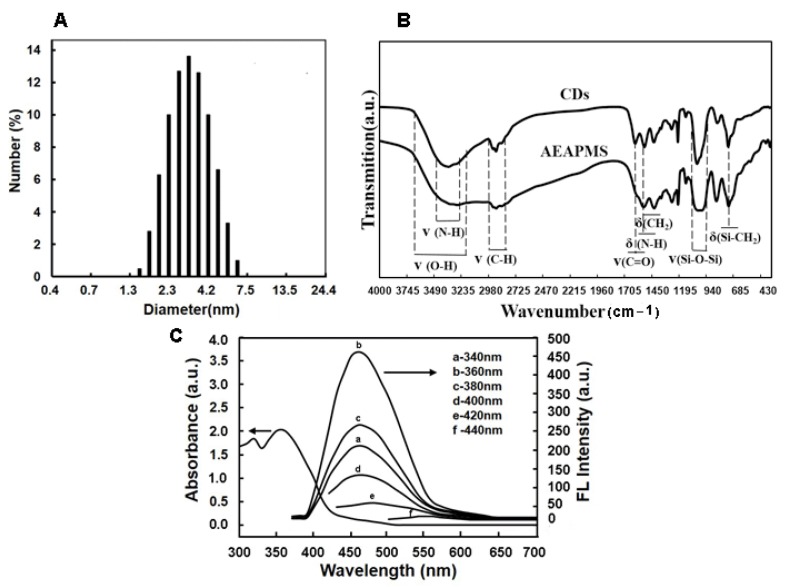
(**A**) Size distribution of CDs (obtained from the DLS measurement in ethanol); (**B**) FT-IR spectra of AEAPMS and CDs; (**C**) Absorption and PL emission spectra of the CDs in ethanol.

Multi-aminosilane (AEAPMS) modified CDs with a highly fluorescent property were prepared by an efficient one-step reaction and characterized by FT-IR, DLS, and fluorescence spectroscopy, respectively. As demonstrated in Figure 2A, CD microspheres with a well-distributed size were obtained, with an average particle size of 3.4 nm. As reported, during CD synthesis, a surface passivation reaction occurred between the amine groups of AEAPMS and the carboxyl groups derived from the pyrolysis of anhydrous citric acid [36]. Therefore, the acylation was confirmed by FT-IR spectroscopy (Figure 2B). Different from AEAPMS, the spectrum of CDs exhibited two characteristic peaks at 1650 cm^−1^ and 1565 cm^−1^, which were similar to the vibrations of amide I and II, respectively. These typical signals of C=ONR vibrations indicated the successful passivation reaction on the CD surface. In addition, the CDs showed an evident adsorption peak at 360 nm (Figure 2C). When excited in the spectrum of 340–400 nm, the CDs showed strong blue PL at 460 nm, which was strongly dependent on the excitation energy.

### 2.2. MIFQP Preparation and Characterization

**Figure 3:**
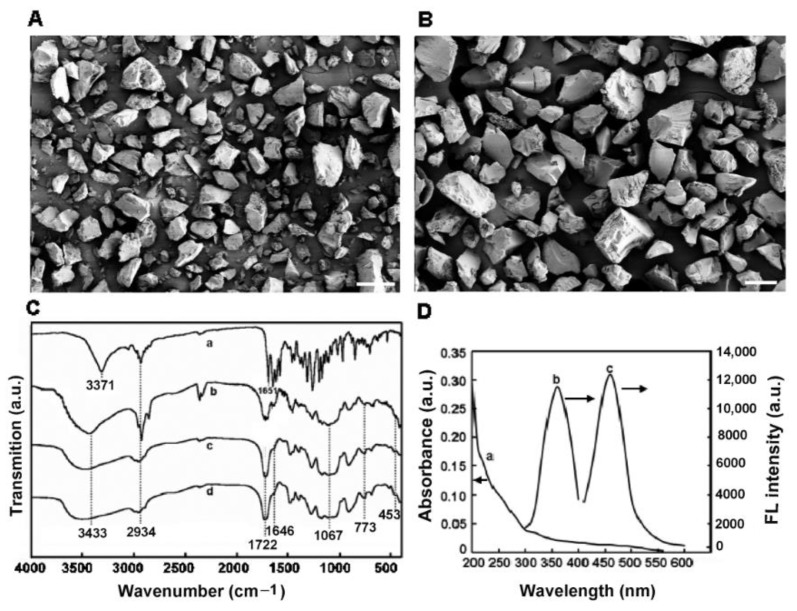
SEM images of (**A**) MIFQP and (**B**) control; (**C**) FT-IR spectra of (a) ZEA, MIFQP (b) before and (c) after extraction, and (d) control polymer after extraction; (**D**) (a) UV-vis absorption, (b) FL excitation, and (c) emission spectra of MIFQP. Scale bar = 100 μm.

A ZEA imprinted fluorescence quenching mesoporous structured polymer encapsulated with CDs was prepared by the NHSG route in this study, where ZEA, methacrylic acid (MAA), azobisisobutyronitrile (AIBN), and γ-methacryloxypropyltrimethoxysilane (MPTMS) acted as the template molecule, functional monomer, free radical initiator, and cross-linker, respectively. Briefly, MAA catalyzed the condensation reaction between MPTMS and the passivated CDs, and then an Si-O-Si framework was formed. Simultaneously, with the thermal initiation of AIBN, carbon-carbon double bonds between MAA and MPTMS polymerized. Consequently, CDs with high luminescence were encapsulated in the silica-based materials. After eluting the template molecule by acetone, the MIFQP with specific recognition sites for ZEA was generated. As shown in Figure 3A,B, both MIFQP and a control polymer exhibited a similar rough surface, with a narrowly distributed particle size around 100 μm. FT-IR spectra of the imprinted and control polymer further confirmed that ZEA was successfully embedded into the MIFQP (Figure 3C). In detail, the strong broad peaks at 3371 cm^−1^ and 1651 cm^−1^ resulted from the stretching vibration of hydroxyl and carbonyl groups of the ZEA molecule (Figure 3C-a). These peaks were also obvious for MIFQP before extraction (Figure 3C-b), but disappeared after the extraction of ZEA (Figure 3C-c). Therefore, after polymerization, ZEA was successfully grafted into the silica matrix, and could be washed out with a suitable solvent. In addition, the strong peak around 1067 cm^−1^ was ascribed to the Si-O-Si asymmetric stretching, and Si-O vibrations showed characteristic peaks at 773 cm^−1^ and 453 cm^−1^. All of these characteristics further confirmed that through NHSG condensation of the silane reagents, CDs were successfully encapsulated into the imprinted polymer. Besides, similar FT-IR spectra between extracted MIFQP and control polymers indicated that they have a similar composition, and the template molecules had been removed with extraction (Figure 3C-c,d). Furthermore, in order to confirm the successful encapsulation of CDs into the MIP, the fluorescence excitation and emission spectra of MIFQP were tested. The results showed that, consistent with the results demonstrated in Figure 2C, when excited at 360 nm, the synthesized MIFQP showed an obvious fluorescence emission peak at 460 nm (Figure 3D), suggesting that the fluorescence properties of CDs were not influenced during the preparation process due to the optically transparent and inert property of silica [37].

**Figure 4:**
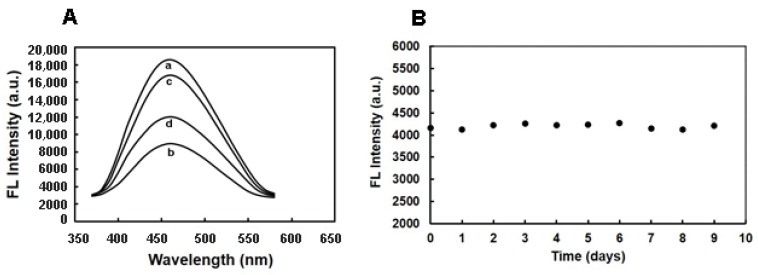
(**A**) FL spectra of (a) control polymer, MIFQP (b) before and (c) after removal of template, and (d) MIFQP with addition of ZEA (500 μg mL^−1^ of ZEA in water); (**B**) Stability of fluorescence emission property of MIFQP in water (the concentration of ZEA is 312.5 μg mL^−1^).

The synthesized MIFQP was then evaluated for ZEA sensing. As shown in Figure 4A, after removal of the template (Figure 4A-c), the fluorescence intensity of MIFQP almost reached the same level as the control (non-imprinted polymers) (Figure 4A-a). After the addition of ZEA, the fluorescence of MIFQP was sharply quenched (Figure 4A-d), and the quenching was inversely proportional to the amount of ZEA (Figure 4A). The mechanism of FL quenching may be ascribed to the electron transfer from the acceptor to donor molecules in solution [38,39] in which ZEA and CDs may act as the electron acceptor and donor, respectively. The MIFQP can bind ZEA, and the bound ZEA will accept electrons from CDs in the MIPs; therefore, the FL of MIFQP could be effectively quenched after the incorporation with ZEA molecules. Furthermore, the FL photostability of the polymers was investigated in the study. The result indicated that the fluorescence intensities of MIFQP suspension were stable within 10 consecutive days (standard deviation (RSD) of 1.19%) when the concentration of ZEA was 312.5 μg mL^−1^ (Figure 4B), which indicates the excellent photostability of the obtained MIFQP.

### 2.3. Selectivity and Sensitivity of MIFQP Experiments

**Figure 5:**
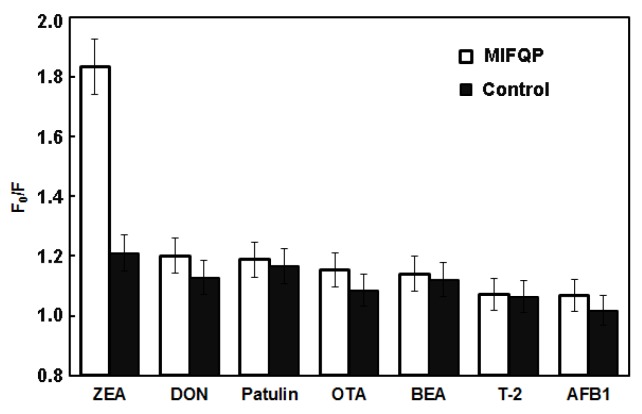
Selectivity of MIFQP and control for ZEA, DON, patulin, OTA, BEA, T-2, and AFB1. The concentration was 250 μg mL^−1^ of each compound.

The selectivity and sensitivity of MIFQP were evaluated by measuring their fluorescent responses to ZEA and other mycotoxins such as AFB1, DON, OTA, T-2, patulin, and BEA (250 μg mL^−1^). As indicated in Figure 5, ZEA could obviously quench the FL of the imprinted polymer (*F*_0_/*F*-1 = 0.834), which was 3.97 times higher than that of the control (non-imprinted polymers). However, for the other compounds, a similar fluorescence change was observed for MIFQP and the control, suggesting that the MIFQP are ZEA specific. Additionally, the fluorescence quenching efficiency of ZEA towards the materials was much higher than other mycotoxins, which indicates the high selectivity of the imprinted polymers. The specificity of MIFQP towards ZEA may be attributed to the shapes of the cavities and presence of imprinted binding sites, which were formed during the polymerization. However, the fluorescence quenching of other compounds was mainly because of the non-specific bindings.

### 2.4. Validation and Application of MIFQP

**Figure 6:**
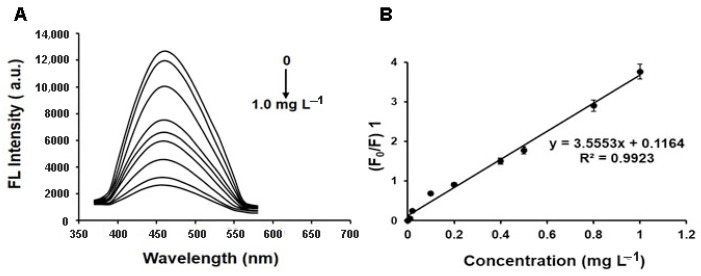
(**A**) MIFQP upon addition of the indicated concentration of ZEA; (**B**) Calibration curves of MIFQP for ZEA.

In order to evaluate the applicability of the proposed polymer for ZEA determination in real samples, we added ZEA into the raw corn samples before sample preparation, and then analyzed the spiked samples. As indicated in the figure, with increasing ZEA concentrations, the fluorescence intensity gradually decreases for MIFQP. The fluorescence quenching in this system was in accordance with the Stern-Volmer equation [40], *F*_0_/*F* = 1 + *K*_sv_ *C*_ZEA_, where *F*_0_ and *F* represent the fluorescence intensities without or with the template (quencher), respectively; *K*_sv_ is the quenching constant of the quencher; and *C*_ZEA_ is the concentration of quencher. As demonstrated in Figure 6A, the MIFQP exhibited an obviously higher fluorescence quenching efficiency. As shown in Figure 6B, the signal (*F*_0_/*F*-1) of the assay exhibited a linear correlation to ZEA concentration. In detail, the linear plots of fluorescence quenching of MIFQP versus ZEA concentrations were depicted within the concentration range of 0.02–1.0 mg L^−1^, with a correlation coefficient of 0.9923. The LOD of ZEA for MIFQP was 20 μg L^−1^ and the LOQ was 60 μg L^−1^ (*S*/*N* = 3), which is higher than the ELISA or HPLC-MS/MS, but is far below the maximum limits (MLs) (200 μg kg^−1^) of ZEA in a cereal sample set by the Europe Union [41]. At three spiking concentrations (200, 400, and 800 μg kg^−1^), the recoveries ranged from 78% to 105%, with the RSD lower than 20% (Table 1).

Once the method was validated, MIFQP sensors were applied to investigate the occurrence of ZEA in 22 corn samples. The results showed that 77% (17/22) of samples were found to be positive for ZEA. Among these ZEA contaminated samples, four were contaminated above the MLs. Therefore, the ZEA specific MIFQP biosensor developed in the study is promising for actual applications.

## 3. Conclusions

In summary, a one-step method for the preparation of hydrophilic, highly sensitive, and selective fluorescence quenching materials was developed in this study. The FL sensor showed high specificity and excellent optical readout. The MIFQP was confirmed to be applicable for ZEA determination in corn samples. In considering the simple synthesis of particles and their excellent dispersion and fluorescence properties in aqueous solution, we believe that such polymers may be promising in the analysis of other contaminants.

## 4. Materials and Methods

### 4.1. Materials

N-(β-aminoethyl)-γ-aminopropylmethyldimethoxysilane (AEAPMS, 97%), azobisisobutyronitrile (AIBN, 99%), methacrylic acid (MAA, 99%), γ-methacryloxypropyltrimethoxysilane (MPTMS, 99%), and anhydrous citric acid (99%) were purchased from Sigma-Aldrich (St. Louis, MO, USA, https://www.sigmaaldrich.com/). Acetonitrile and methanol were obtained from Merck (Darmstadt, Germany, http://www.merckmillipore.com/). Zearalenone (ZEA, 99%), deoxynivalenol (DON, 99%), ochratoxin (OTA, 99%), aflatoxin B1 (AFB1, 99%), patulin (99%), beauvericin (BEA, 99%), and T-2 (99%) were from Fermentek (Jerusalem, Israel, https://www.fermentek.com/). Acetic acid (99%) and other chemical reagents were provided by Sinopharm Co. (Shanghai, China, http://www.sinopharmholding.com/en/). All reagents were of analytical grade.

### 4.2. Instruments and Measurements

FL measurements were performed with an Infinite M200 PRO instrument (TECAN, Switzerland, https://www.tecan.com/), while UV-vis spectra analyses were performed on a NanoDrop 2000 Spectrophotometer (https://www.thermofisher.com). Fourier transform infrared (FT-IR) spectroscopic tests were performed on a Bio-Rad FTS6000 spectrophotometer (www.bio-rad.com/). Scanning electron microscopy (SEM), Hitachi SU1510, was used to characterize the surface morphologies of MIPs and NIPs (https://www.hitachi-hightech.com). A dynamic laser scattering (DLS) spectrometer (Zetasizer Nano ZS90, https://www.malvernpanalytical.com) was used to determine the size distribution of the MIFQP.

### 4.3. CDs and MIFQP Synthesis

CDs were synthesized according to Wang et al. [34]. Briefly, AEAPMS (10 mL) was put into a 100-mL three-necked flask and degassed for 5 min with nitrogen. Then, the flask was heated to 240 °C, and 0.5 g citric acid anhydrous was quickly added, followed by vigorous stirring. After cooling to room temperature, the synthesized materials were purified three times with petroleum ether. Afterwards, the polymer was produced via an NHSG process. A total of 0.05 mmol ZEA, 100 µL CDs, and 2.0 mmol MAA were sequentially dissolved in a solution of 4.0 mL chloroform containing 1.0 mL acetonitrile. The mixture was sealed and stirred in the dark for 1 h. Then, 3.0 mmol MPTMS and 20.0 mg AIBN were added. Before being sealed, the mixture was sonicated for 10 min and purged with oxygen-free nitrogen for 5 min. Finally, the polymerization occurred at 55 °C for 18 h in the dark. The resulting bulk polymers were grounded mechanically and wet-sieved with acetone through a 200-mesh steel sieve. After extraction with acetone/acetic acid (8:2, *v*/*v*) for 48 h with a soxhlet apparatus (removal of ZEA), the MIFQP was dried at 60 °C for 12 h. Control polymers were simultaneously prepared as described above without the addition of a ZEA template.

### 4.4. FL Measurements

In order to measure the recognition capacity of polymers and the control, 50 μg of each compound was separately dispersed in 150 μL acetic acid (5%) with different concentrations of ZEA in a Corning Costar 96-Well Black Polystyrene Plate, and shaken for 4 h at room temperature. The adsorption of MIFQP and control was measured by fluorescence spectroscopy with excitation at 360 nm. The excitation and emission slits were 5 nm.

### 4.5. Sample Pretreatment

Maize samples were purchased from local markets in Shanghai, China. For sample preparation, 2 g of finely ground corn and 5 mL water were put into a 50 mL polypropylene centrifuge tube. Then, the tube was vortexed for 1 min, followed by the addition of 5 mL of acidified ACN (1% FA). To completely disperse the sample, the tube was vigorously shaken for 30 min, and then anhydrous MgSO_4_ (1 g) and NaCl (0.25 g) were added, followed by immediate shaking for 30 s and centrifuging for 5 min at 6000× *g*. The supernatant was transferred into another 5 mL tube and evaporated at 40 °C with nitrogen. The residue was then resolved with 1 mL methanol and stored at 4 °C until analysis.

### 4.6. Method Validation and Quality Assurance

A spiked corn sample was used to validate the method. The evaluated parameters included linearity, recovery, repeatability (RSD_r_), reproducibility (RSD_R_), limits of detection (LODs), and limits of quantification (LOQs).

## Figures and Tables

**Table 1:** Overview of the recoveries, repeatability (RSD_r_), reproducibility (RSD_R_), and limits of detection and quantitation (LOD and LOQ) of ZEA in corn determined with MIFQP. (*n* = 3).

Sample	Concentration of ZEA (mg L^−1^)	Recovery (%)	RSD_r_	RSD_R_	LOD (mg L^−1^)	LOQ (mg L^−1^)
1	0.2	105.1	13.3	16.3	0.02	0.06
2	0.4	78.2	10.1	14.1		
3	0.8	90.7	8.7	12.9		
